# The future costs of cancer attributable to excess body weight in Brazil, 2030-2040

**DOI:** 10.1186/s12889-022-13645-4

**Published:** 2022-06-21

**Authors:** Leandro F. M. Rezende, Thainá Alves Malhão, Rafael da Silva Barbosa, Arthur Orlando Correa Schilithz, Ronaldo Corrêa Ferreira da Silva, Luciana Grucci Maya Moreira, Paula Aballo Nunes Machado, Bruna Pitasi Arguelhes, Maria Eduarda Leão Diogenes Melo

**Affiliations:** 1 Department of Preventive Medicine, Escola Paulista de Medicina, Universidade Federal de São Paulo, São Paulo, Brazil; 2 Cancer Prevention and Surveillance Coordination Unit, Brazilian National Cancer Institute José Alencar Gomes da Silva, Rio de Janeiro, Brazil; 3 Postgraduate Program in Social Policy, Federal University of Espírito Santo, Vitoria, Brazil; 4 Department of Basic and Experimental Nutrition, Nutrition Institute, State University of Rio de Janeiro, Rio de Janeiro, Brazil

**Keywords:** Cancer, Overweight, Obesity, Cost of cancer, Cost-of-illness economic burden of diseases

## Abstract

**Background:**

Excess body weight (EBW), herein defined as body mass index (BMI) ≥25 kg/m^2^, is a well-known modifiable risk factor for cancer and a pivotal vector for growing healthcare costs. We estimated the future (2030) federal direct healthcare costs of cancer in the Brazilian Unified Health System (SUS) attributable to EBW. We also projected direct healthcare costs of cancer that could be potentially saved in 2040, considering counterfactual (alternative) scenarios of population-wide reductions in the BMI to be achievedin 2030.

**Methods:**

We developed a macrosimulation model by sex using self-reported BMI data in adults ≥ 20 years who relied exclusively on the public health system from the Brazilian National Health Survey (PNS) 2019; relative risks for 12 types of cancer from the World Cancer Research Fund/American Institute Cancer Research (WCRF/AICR) meta-analysis; and nationwide registries of federal direct healthcare costs of inpatient and outpatient procedures in adults ≥30 years with cancer from 2008-2019. We calculated the attributable costs of cancer via comparative risk assessment, assuming a 10-year lag between exposure and outcome. We used the potential impact fraction (PIF) equation and the Monte Carlo simulation method to estimate the attributable costs and 95% uncertainty intervals, considering the theoretical-minimum-risk exposure and other counterfactual (alternative) scenarios of the EBW prevalence. We assessed the cancer costs attributable to EBW, multiplying PIF by the direct healthcare costs of cancer.

**Results:**

In 2030, 2.4% or US$ 62.8 million in direct healthcare costs of cancer may be attributable to EBW. We projected potential savings of approximately US$ 10.3 to 26.6 million in 2040 by reducing the prevalence of EBW in 2030.

**Conclusions:**

We estimated high future costs of cancer attributable to EBW in Brazil. Our findings may support interventions and policies focused on the primary prevention of EBW and cancer.

**Supplementary Information:**

The online version contains supplementary material available at 10.1186/s12889-022-13645-4.

## Background

The prevalence of overweight and obesity has increased over the last decades in Brazil, as in other countries. Between 2008/09 and 2019, the prevalence of excess body weight (EBW, herein defined as body mass index - BMI ≥25 Kg/m^2^) increased by 20% (from 50.1 to 60.3%), whereas the prevalence of obesity (BMI ≥30 Kg/m^2^) more than doubled in the same period (from 12.5 to 25.9%) [1]. The increase in the prevalence of EBW will lead to further increases in the burden of non-communicable diseases, such as diabetes, cardiovascular diseases, and cancers [2].

The WCRF/AICR considers the evidence strong that EBW increases the risk of several types of cancer, namely breast (postmenopausal); colorectal; endometrium; gallbladder; kidney; liver; mouth, pharynx, and larynx; esophagus (adenocarcinoma); ovary; pancreas; prostate (advanced stage), and stomach (cardia) [3]. In Brazil, there were an estimated 556,995 new cancer cases in 2020 (excluding non-melanoma skin cancer), of which 64% (*n* = 357,468) were EBW-related cancers (breast (any): *n* = 88,492; colorectal: *n* = 55,102; endometrium: *n* = 11,791; gallbladder: *n* = 2,027; kidney: *n* = 11,971; liver: *n* = 12,674; mouth, pharynx, and larynx: *n* = 27,026; esophagus (any): *n* = 10,363; ovary: *n* = 7,298; pancreas: *n* = 13,307; prostate (any): *n* = 97,278; and stomach (any): *n* = 20,139) [4]. Additionally, EBW was estimated to account for more than 15 thousand (4%) new cancer cases in Brazil in 2012 and could cause over 29 thousand cancers per year (5%) in 2025 [5].

Primary cancer prevention is imperative to cope with the increasing burden of cancer, particularly in settings with limited access to affordable and effective cancer treatment, such as low- and middle-income countries. It is important to note that the prohibitive’ increase in cancer treatment costs is a concern. We previously estimated the current (2018) federal direct healthcare cost of cancer in the SUS attributable to EBW [6]. In 2018, the Brazilian federal direct healthcare costs of cancer were Int $1.7 billion, of which EBW was estimated to account for 1.8% or Int$ 30 million [6]. In addition to the current cost-of-illness attributable to EBW, quantifying the projected future economic burden of cancer attributable to EBW may inform the potential impact of policies and public health interventions.

Herein, we estimated the future (2030) federal direct healthcare costs of cancer in the SUS attributable to EBW. In addition, we estimated potential savings in federal direct healthcare costs of cancer in 2040 by considering different counterfactual (alternative) scenarios of reduction in the prevalence of EBW to be achieved in 2030.

## Methods

### Data and study design

This study applied a top-down costing methodology and performed a macrosimulation model to estimate the future costs of cancer attributable to EBW, using the Brazilian population as a case study. We used the following data: 1. Relative risks from WCRF/AICR meta-analyses (Supplementary Material A); 2. Prevalence data (%) of BMI categories in adults aged 20 years or older who relied exclusively on the public health system from the PNS carried out in 2019; 3. Nationwide registries of federal direct healthcare costs of inpatient and outpatient procedures in the SUS in adults aged 30 years or older with cancer. Parameters used in the model are available in Supplementary Material B.

We estimated the impact of EBW on federal direct healthcare costs of cancer, assuming a 10-year time lag between exposure and outcome via comparative risk assessment. We used the potential impact fraction (PIF) equation and the Monte Carlo simulation method to estimate the attributable costs and their 95% uncertainty intervals, considering the theoretical-minimum-risk exposure and other counterfactual (alternative) scenarios of the EBW prevalence. We assessed the cancer costs attributable to EBW, multiplying PIF by the direct healthcare costs of cancer.

### Relative risk estimates and cancer sites

We included cancer sites with strong evidence of association (convincing or probable) with EBW according to the WCRF/AICR [3]. We detailed the list of the 10^th^ Revision of the International Statistical Classification of Diseases and Related Health Problems (ICD-10) codes in Supplementary Material C.

We obtained the relative risks (${RR}_{x}$) for EBW-associated cancers incidence by sex from the WCRF/AICR dose-response meta-analysis (Supplementary Material A), considering the increment of $x$ kg/m^2^ of BMI ($x$ = 5)). For prostate (advanced) cancer and ovary cancer, the summary ${RR}_{x}$ included studies with mortality and incidence outcomes. For this reason, we reperformed the WCRF/AICR meta-analysis using random-effects models considering only the incidence as the outcome (Supplementary Material D and E).

We converted these measures per increment of 1 kg/m^2^ of BMI (${RR}_{1}$) using the following equation [7]:

$${RR}_{1}=exp(\frac{\mathrm{log}({RR}_{x})}{x}).$$

To obtain the RR for each BMI category (RRc) (Supplementary Material F), we used the following equation [8]:

$${RR}_{c}={RR}_{1}^{{M}_{c}-ref},$$

where Mc represents the median value in each category, and ref represents the reference category value (< 25 kg/m^2^). The reference category reflected the theoretical minimum risk exposure level [3] and the Brazilian National Cancer Institute recommendations [9].

#### Assessment of EBW prevalence in 2019

We obtained self-reported height and weight from the adult population aged ≥20 years from the PNS 2019 [1], a nationally representative health survey conducted in Brazil. The PNS 2019 microdata are available in the public domain via the Brazilian Institute of Geography and Statistics (IBGE) in partnership with the Ministry of Health at http://www.ibge.gov.br (Supplementary Material G). We considered only adults aged 20 years or older who reported not having health insurance to obtain the prevalence per BMI categories by sex and their corresponding 95% confidence intervals. For postmenopausal breast cancer, we considered women aged 50 years or older. We incorporated the complex sample design into all estimates using RStudio version 1.4.1103.

**Fig. 1:**
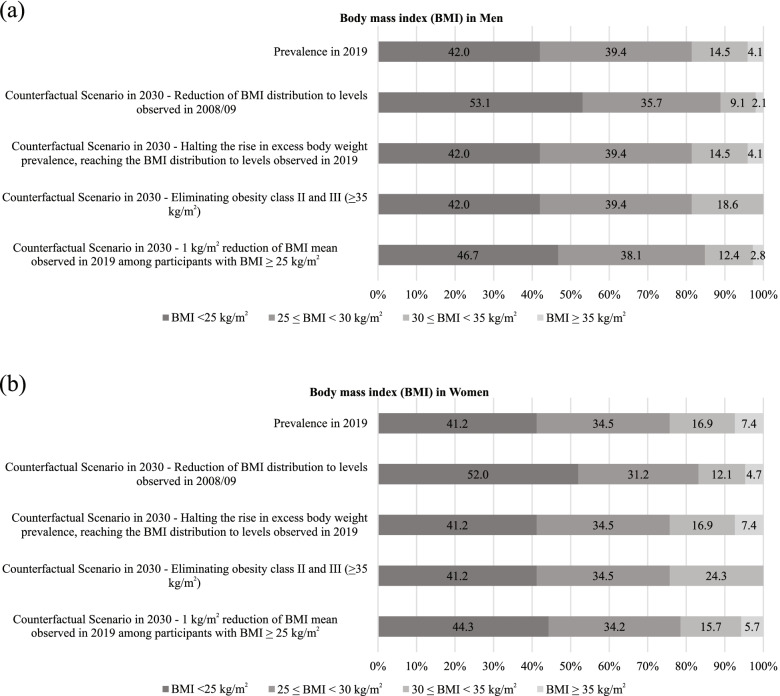
Body mass index distribution in 2019 and levels fixed in counterfactual (alternative) scenarios to be achieved in 2030

We displayed BMI categories in Fig. 1 and Supplementary Material F. The reference category (BMI < 25 kg/m^2^) aimed to reflect the theoretical minimum risk exposure level regarding cancer risk [3] and the recommendations of the Brazilian National Cancer Institute (INCA) [9].

#### Counterfactual (alternative) scenarios for BMI reductions

We proposed four counterfactual (alternative) scenarios of BMI population-wide reductions in 2019 to be achieved in Brazil in 2030 to save direct healthcare costs with cancer in 2040 (Fig. 1). The scenarios considered the following: 1) reduction of BMI distribution to levels observed in the National Household Budget Survey (POF) carried out in 2008/09 [10] (Supplementary Material G); 2) halting the rise in excess body weight prevalence, reaching the BMI distribution to levels observed in 2019; 3) eliminating obesity class II and III (BMI≥35 kg/m^2^); 4) 1 kg/m^2^ reduction of BMI’ mean observed in 2019 among participants with BMI ≥ 25 kg/m^2^ [2]. We based the counterfactual (alternative) scenarios on policy targets [11] and theoretical discussion in the literature [2, 5].

#### Federal direct healthcare costs of cancer in the Brazilian Unified Health System in 2030 and 2040

We retrieved registries of federal direct healthcare costs of inpatient and outpatient procedures related to cancer between 2008 and 2019 from the Hospital Information System (SIH) and Ambulatory Information System (SIA) of the Brazilian Public Health System (SUS) (Supplementary Material G). We used the 10^th^ Revision of the International Statistical Classification of Diseases and Related Health Problems (ICD-10) codes for recovering cancer procedures from information systems (Supplementary Material C). We stratified the direct healthcare costs by sex and cancer type. Assuming a 10-year time lag between exposure and outcome, we considered the procedures approved for payment in adults with cancer aged 30 years or older in 2030 and 2040. For postmenopausal breast cancer, we considered women aged 60 years or older.

We performed a simple linear regression to predict the future costs of each cancer type evaluated (dependent variable) as a function of time (independent variable) up to 2030 and 2040 based on the values practiced over time between 2008 and 2019. It is crucial to control for potential confounders while examining the possible determinants of cost. Once our outcome was the direct healthcare costs over time, it was unnecessary to control for confounders because we observed their effect in the observed costs used to fit the regression model [12]. We transformed the monetary values in Brazilian Real (R$) to United States Dollar (US$), considering the purchasing power parity (PPP) of 2019 (conversion factor 2.281) [13].

#### Cancer cost attributable to EBW

Based on the abovementioned intermediate outputs of the models, we calculated the potential impact fraction (PIF) for EBW-cancers subtypes (Table 1) by sex and counterfactual (alternative) scenario using the following equation [14]:

$$PIF\;subtype = \frac{{\sum\nolimits_{i = 1}^{n} {P_{i} } \;RR_{i} - \sum\nolimits_{i = 1}^{n} {P^{\prime}_{i} } \;RR_{i} }}{{\sum\nolimits_{i = 1}^{n} {P_{i} } \;RR_{i} }},$$

**Table 1:** Relative risks and potential impact fraction^a^ by sex and type of cancer

**Cancer type**	**Sex**	**RR per 5 kg/m**^**2**^	**RR per 1 kg/m**^**2**^	**PIF subtype%**
Breast (post-menopausal)	F^b^	1.12 (1.09-1.15)	1.02 (1.02-1.03)	6.83 (5.25-8.39)
Colorectal	F^b^	1.05 (1.02-1.08)	1.01 (1.00-1.02)	2.81 (1.16-4.45)
	M^b^	1.08 (1.04-1.11)	1.02 (1.01-1.02)	3.60 (1.95-5.15)
Endometrium	F^b^	1.50 (1.42-1.59)	1.08 (1.07-1.10)	24.60 (23.07-26.09)
Esophagus (adenocarcinoma)	F^b^	1.48 (1.29-1.71)	1.08 (1.05-1.11)	23.75 (21.54-25.95)
	M^b^	1.56 (1.39-1.74)	1.09 (1.07-1.12)	21.78 (19.79-23.73)
Gallbladder	F^b^	1.23 (1.10-1.39)	1.04 (1.02-1.07)	12.29 (10.14-14.40)
	M^b^	1.23 (1.10-1.39)	1.04 (1.02-1.07)	9.84 (7.61-11.96)
Kidney	F^b^	1.30 (1.25-1.36)	1.05 (1.05-1.06)	15.68 (14.12-17.20)
	M^b^	1.30 (1.25-1.36)	1.05 (1.05-1.06)	12.55 (10.98-14.10)
Liver	F^b^	1.43 (1.19-1.70)	1.07 (1.04-1.11)	21.60 (18.97-24.21)
	M^b^	1.43 (1.19-1.70)	1.07 (1.04-1.11)	17.33 (14.60-20.01)
Mouth, Pharynx and Larynx	F^b^	1.15 (1.06-1.24)	1.03 (1.01-1.04)	8.22 (6.36-10.04)
	M^b^	1.15 (1.06-1.24)	1.03 (1.01-1.04)	6.59 (4.66-8.41)
Ovary	F^c^	1.06 (1.02-1.10)	1.01 (1.00-1.02)	3.17 (1.49-4.80)
Pancreas	F^b^	1.10 (1.04-1.16)	1.02 (1.01-1.03)	5.57 (3.79-7.26)
	M^b^	1.13 (1.04-1.22)	1.02 (1.01-1.04)	5.74 (3.89-7.60)
Prostate (advanced)	M^c^	1.08 (1.03-1.14)	1.02 (1.01-1.03)	3.70 (2.02-5.35)
Stomach (cardia)	F^b^	1.23 (1.07-1.40)	1.04 (1.01-1.07)	12.30 (9.92-14.57)
	M^b^	1.23 (1.07-1.40)	1.04 (1.01-1.07)	9.84 (7.49-12.23)

Between parenthesis: 95% confidence intervals for RR and 95% uncertainty intervals for PIF

*Abbreviations*: *RR* Relative risk, *PIF* Potential impact fraction, *F* Female, *M* Male

^a^Considering the theoretical minimum risk exposure level

^b^RR was calculated by meta-analysis studies from WCRF/AICR SLR CUP Reports (Supplementary Information A)

^c^RR was calculated by meta-analysis studies excluding mortality studies from WCRF/AICR SLR CUP Reports (Supplementary Information D and E)

where ${P}_{i}$ is the proportion of the population at the level $i$ of BMI category in a given year, $P^{\prime}_{i}$ is the proportion of the population at the level $i$ of BMI category in a given counterfactual (alternative) scenario, and ${RR}_{i}$ is the relative risk of cancer subtype (whenever appropriated) at the level $i$ of BMI. We displayed the levels $i$ for the BMI category in Supplementary Information B. Of note, the PIF equals the Population Attributable Fraction (PAF) when the counterfactual (alternative) scenario represents the theoretical minimum risk exposure level.

Decision-makers may be more interested in cost by topography (Tables 2 and 3) rather than cancer subtype. Therefore, for esophagus (adenocarcinoma), prostate (advanced), stomach (cardia), and postmenopausal breast cancer, we recalculated the PIF by the topographic site: esophagus, prostate, stomach, and breast (women aged 30 years or older), using the following equation:

**Table 2:** Future (2030) federal direct healthcare costs (in Million US$)^a^ of cancer attributable to excess body weight ^b^ in Brazil

**Cancer type**	**Sex**	**PIF type**^**c**^	**Cancers costs**^**a**^	**Attributable costs**
		**(%)**	**(in Million US$)**	
Breast (any)	F	2.70 (2.08-3.32)	620.39	16.77 (12.89-20.60)
Colorectal	F	2.83 (1.19-4.45)	220.93	6.26 (2.63-9.83)
	M	3.58 (1.92-5.17)	228.91	8.19 (4.39-11.83)
	T	3.21 (1.56-4.81)	449.84	14.44 (7.02-21.66)
Endometrium	F	24.60 (23.07-26.10)	36.62	9.01 (8.45-9.56)
Esophagus (any)	F	2.28 (2.06-2.49)	9.76	0.22 (0.20-0.24)
	M	1.88 (1.70-2.04)	34.56	0.65 (0.59-0.71)
	T	1.96 (1.78-2.14)	44.32	0.87 (0.79-0.95)
Gallbladder	F	12.28 (10.20-14.43)	2.31	0.28 (0.24-0.33)
	M	9.86 (7.66-11.99)	1.14	0.11 (0.09-0.14)
	T	11.48 (9.36-13.62)	3.45	0.40 (0.32-0.47)
Kidney	F	15.67 (14.13-17.19)	8.71	1.36 (1.23-1.50)
	M	12.57 (11.00-14.11)	12.88	1.62 (1.42-1.82)
	T	13.82 (12.26-15.35)	21.59	2.98 (2.65-3.31)
Liver	F	21.61 (19.01-24.20)	5.48	1.18 (1.04-1.33)
	M	17.32 (14.60-20.06)	8.79	1.52 (1.28-1.76)
	T	18.97 (16.30-21.65)	14.27	2.71 (2.32-3.09)
Mouth, Pharynx and Larynx	F	8.21 (6.37-10.07)	20.42	1.68 (1.30-2.06)
	M	6.57 (4.68-8.40)	92.01	6.04 (4.31-7.73)
	T	6.87 (4.99-8.70)	112.43	7.72 (5.61-9.78)
Ovary	F	3.17 (1.46-4.87)	61.78	1.96 (0.90-3.01)
Pancreas	F	5.57 (3.79-7.25)	20.77	1.16 (0.79-1.51)
	M	5.72 (3.79-7.58)	20.08	1.15 (0.76-1.52)
	T	5.65 (3.79-7.41)	40.85	2.31 (1.55-3.03)
Prostate (any)	M	0.91 (0.49-1.31)	352.87	3.21 (1.72-4.63)
Stomach (any)	F	0.47 (0.38-0.56)	26.58	0.13 (0.10-0.15)
	M	0.56 (0.43-0.70)	47.55	0.27 (0.20-0.33)
	T	0.53 (0.41-0.65)	74.13	0.39 (0.31-0.48)
EBW - associated cancer	F	3.87 (2.88-4.85)	1 033.74	40.01 (29.76-50.09)
	M	2.85 (1.85-3.81)	798.77	22.76 (14.77-30.46)
	T	3.43 (2.43-4.40)	1 832.52	62.77 (44.53-80.56)
All invasive cancers^d^	F	2.76 (2.05-3.45)	1 450.00	40.01 (29.76-50.09)
	M	2.00 (1.30-2.68)	1 137.20	22.76 (14.77-30.46)
	T	2.43 (1.72-3.11)	2 587.20	62.77 (44.53-80.56)

Between parenthesis: 95% uncertainty intervals

*Abbreviations*: *PIF* Potential impact fraction, *F* Female, *M* Male, *T* Both sexes, *EBW* Excess body weight

^a^Projected direct healthcare costs (inpatient and outpatients) of cancer in adults ≥30 years

^b^Defined as Body Mass Index – BMI ≥25 Kg/m^2^

^c^Considering the theoretical minimum risk exposure level

^d^Codes of the 10^th^ revision of the International Statistical Classification of Diseases and Related Health Problems: C00-C97

**Table 3:** Potential impact of reduction in excess body weight^a^ on costs of cancer in 2040

**Cancer type**	**Sex**	**Projected costs**^**b**^	**Scenario 1**^**c**^	**Scenario 2**^**d**^	**Scenario 3**^**e**^	**Scenario 4**^**f**^
		**(in million US$)**				
Breast (any)	F	834,70 (763,84-905,55)	4.60 (0.00-10.31)	6.36 (0.87-11.63)	3.26 (0.00-8.61)	2.29 (0.00-7.58)
Colorectal	F	305.06 (270.74-339.38)	2.08 (0.00-7.08)	2.19 (0.00-7.05)	1.30 (0.00-6.06)	0.91 (0.00-5.72)
	M	317.43 (283.18-351.68)	5.14 (0.07-10.35)	3.46 (0.00-8.30)	1.20 (0.00-6.08)	1.67 (0.00-6.73)
	T	622.49 (553.92-691.06)	7.22 (0.00-17.43)	5.65 (0.00-15.35)	2.50 (0.00-12.14)	2.58 (0.00-12.45)
Endometrium	F	50.08 (43.35-56.80)	3.07 (2.09-4.04)	3.37 (2.52-4.21)	2.71 (1.81-3.56)	1.44 (0.55-2.31)
Esophagus (any)	F	12.47 (9.86-15.08)	0.07 (0.05-0.09)	0.08 (0.06-0.10)	0.06 (0.04-0.08)	0.03 (0.01-0.05)
	M	44.90 (36.06-53.74)	0.43 (0.35-0.51)	0.27 (0.21-0.34)	0.14 (0.07-0.20)	0.13 (0.07-0.20)
	T	57.37 (45.92-68.82)	0.50 (0.40-0.60)	0.35 (0.26-0.44)	0.20 (0.11-0.28)	0.17 (0.08-0.25)
Gallbladder	F	2.91 (1.87-3.95)	0.09 (0.03-0.14)	0.09 (0.05-0.14)	0.06 (0.01-0.11)	0.04 (0.00-0.09)
	M	1.52 (1.00-2.03)	0.07 (0.04-0.10)	0.05 (0.02-0.07)	0.02 (0.00-0.04)	0.02 (0.00-0.05)
	T	4.42 (2.87-5.98)	0.16 (0.08-0.23)	0.14 (0.07-0.21)	0.08 (0.01-0.15)	0.06 (0.00-0.13)
Kidney	F	12.28 (10.82-13.74)	0.47 (0.25-0.69)	0.52 (0.32-0.71)	0.36 (0.15-0.56)	0.22 (0.00-0.42)
	M	18.22 (16.30-20.14)	1.10 (0.78-1.42)	0.72 (0.42-1.01)	0.30 (0.00-0.59)	0.35 (0.05-0.64)
	T	30.50 (27.12-33.88)	1.56 (1.04-2.11)	1.23 (0.74-1.72)	0.66 (0.16-1.15)	0.56 (0.06-1.06)
Liver	F	7.72 (6.70-8.75)	0.41 (0.26-0.56)	0.46 (0.32-0.59)	0.35 (0.20-0.49)	0.19 (0.05-0.32)
	M	12.49 (10.45-14.53)	1.08 (0.84-1.33)	0.69 (0.47-0.90)	0.32 (0.11-0.53)	0.34 (0.13-0.55)
	T	20.22 (17.15-23.28)	1.49 (1.10-1.89)	1.15 (0.79-1.49)	0.67 (0.31-1.02)	0.53 (0.18-0.87)
Mouth, Pharynx and Larynx	F	26.63 (21.98-31.28)	0.52 (0.07-0.96)	0.57 (0.14-1.01)	0.36 (0.00-0.79)	0.24 (0.00-0.67)
	M	120.90 (96.39-145.42)	3.64 (1.64-5.74)	2.42 (0.47-4.32)	0.91 (0.00-2.78)	1.13 (0.00-3.06)
	T	147.53 (118.37-176.70)	4.16 (1.72-6.70)	3.00 (0.61-5.34)	1.27 (0.00-3.57)	1.37 (0.00-3.73)
Ovary	F	83.18 (64.30-102.06)	0.60 (0.00-2.01)	0.68 (0.00-2.00)	0.41 (0.00-1.69)	0.26 (0.00-1.59)
Pancreas	F	28.44 (25.97-30.92)	0.37 (0.00-0.86)	0.41 (0.00-0.87)	0.25 (0.00-0.71)	0.16 (0.00-0.63)
	M	27.43 (24.68-30.18)	0.72 (0.27-1.17)	0.48 (0.04-0.91)	0.17 (0.00-0.61)	0.23 (0.00-0.66)
	T	55.87 (50.64-61.10)	1.09 (0.17-2.03)	0.89 (0.00-1.78)	0.42 (0.00-1.32)	0.39 (0.00-1.29)
Prostate (any)	M	475.81 (400.75-550.87)	1.95 (0.08-3.87)	1.31 (0.00-3.15)	0.49 (0.00-2.27)	0.63 (0.00-2.43)
Stomach (any)	F	35.00 (31.05-38.95)	0.03 (0.01-0.04)	0.03 (0.01-0.04)	0.02 (0.00-0.03)	0.01 (0.00-0.03)
	M	62.44 (53.83-71.06)	0.13 (0.08-0.18)	0.09 (0.04-0.13)	0.03 (0.00-0.08)	0.04 (0.00-0.09)
	T	97.44 (84.87-110.01)	0.16 (0.09-0.22)	0.12 (0.05-0.17)	0.05 (0.00-0.11)	0.05 (0.00-0.11)
EBW - associated cancer	F	1 398.47 (1 250.48-1 546.46)	12.30 (1.83-26.79)	14.76 (0.83-28.34)	9.13 (0.00-22.69)	5.78 (0.00-19.40)
	M	1 081.14 (922.64-1 239.65)	14.25 (4.17-24.67)	9.48 (0.00-19.13)	3.58 (0.00-13.18)	4.55 (0.00-14.40)
	T	2 479.61 (2 173.12-2 786.11)	26.55 (2.33-51.46)	24.24 (0.39-47.47)	12.71 (0.00-35.87)	10.33 (0.00-33.80)
All invasive cancers^g^	F	1 930.44 (1 743.55-2 117.32)	12.30 (1.83-26.79)	14.76 (0.83-28.34)	9.13 (0.00-22.69)	5.78 (0.00-19.40)
	M	1 506.06 (1 324.66-1 687.46)	14.25 (4.17-24.67)	9.48 (0.00-19.13)	3.58 (0.00-13.18)	4.55 (0.00-14.40)
	T	3 436.50 (3 068.20-3 804.79)	26.55 (2.33-51.46)	24.24 (0.39-47.47)	12.71 (0.00-35.87)	10.33 (0.00-33.80)

Between parenthesis: 95% uncertainty intervals

*Abbreviations*: *F* Female, *M* Male, *T* Both sexes, *EBW* Excess body weight

^a^Defined as Body Mass Index – BMI ≥25 Kg/m^2^

^b^Projected direct healthcare costs (inpatient and outpatients) of cancer in adults ≥30 years

^c^Scenario 1: Reduction of BMI distribution to levels observed in the National Household Budget Survey (POF) carried out 2008/09

^d^Scenario 2: Halting the rise in excess body weight prevalence, reaching the BMI distribution to levels observed in 2019

^e^Scenario 3: Eliminating obesity class II and III (≥35 kg/m^2^)

^f^Scenario 4: 1 kg/m^2^ reduction of BMI’ mean observed in 2019 among participants with BMI ≥ 25 kg/m^2^

^g^Codes of the 10^th^ revision of the International Statistical Classification of Diseases and Related Health Problems: C00-C97

$$PIF\;site=\frac{Attributable\;cost\;according\;to\;WCRF\;cancer\;type}{Total\;cost\;of\;topographic\;site}$$

We assessed the cancer costs attributable to EBW, multiplying PIF by the cancer costs. We considered the prevalence in 2019 and the costs of cancer in 2030, assuming at least a 10-year time lag between exposure and outcome (i.e., based on the average follow-up time of prospective cohort studies [3]. Finally, we calculated the potential savings in cancer costs in 2040 if reductions in BMI occurred in Brazil to levels fixed in the counterfactual (alternative) scenarios in 2030.

We quantified the uncertainty in all modeled estimates using the Monte Carlo simulation approach [15, 16] with 10,000 iterations. The simulation works thorough producing a draw from the distributions of a) baseline prevalence per BMI category considering a binomial distribution; b) the log of the relative risks per exposition category for the association between EBW and cancer incidence considering a normal distribution. We calculated PIF by sex for the 50^th^, 2.5^th^, and 97.5^th^ percentiles as the central estimate and 95% uncertainty intervals across all simulations. Negative values of PIF derived from the Monte Carlo simulation were rounded to 0, assuming that reducing BMI values may not increase the risk of cancer and consequently the attributable costs. We used R Studio version 1.3.1093 for analysis.

## Results

### Projected federal direct healthcare costs of cancer in Brazil in 2030 attributable to EBW in 2019

In 2030, we projected approximately US$ 2.6 billion federal direct healthcare costs of cancer in Brazil, of which US$ 1.8 billion were estimated to be EBW-related cancers. We estimated that 2.4% (US$ 62.8 million) of federal direct healthcare costs of cancer might be attributable to EBW. Cancers with the highest PIF were estimated to be endometrium (24.6%), followed by liver (19.0%) and kidney (13.8%). Cancers with the highest attributable costs were estimated to be breast (US$ 16.8 million), followed by colorectal (US$ 14.4 million) and endometrium (US$ 9.0 million) (Tables 1 and 2).

### The potential impact of the reduction in the prevalence of EBW on projected direct healthcare costs of cancer in Brazil in 2040

We displayed in Fig. 1 the potential reductions (counterfactual/ alternative scenarios) in the prevalence of EBW in Brazil to be achieved in 2030. We projected potential savings of approximately US$ 10.3 to 26.6 million in 2040 by reducing the prevalence of EBW (Table 3). The counterfactual (alternative) scenario with the highest potential impact on cancer costs was the reduction of BMI distribution to levels observed in 2008/09 (US$ 26.55 million), followed for the halting the rise in EBW prevalence, reaching the BMI distribution to levels observed in 2019 (US$ 24.24 million), eliminating obesity class II and III (US$ 12.71 million) and 1 kg/m^2^ reduction of BMI’ mean observed in 2019 among participants with BMI ≥ 25 kg/m^2^ (US$ 10.33 million).

## Discussion

Our study showed that overweight and obesity may be responsible for US$ 62.8 million federal direct healthcare costs of cancer in 2030, considering the increasing prevalence of overweight and obesity in the last decade, as well as the rising economic burden of cancers in Brazil. Population-wide reductions of BMI to levels fixed in the counterfactual (alternative) scenarios in 2030 could save US$ 10.3 to 24.2 million direct healthcare costs of cancer in 2040.

The detrimental health effects of EBW have been extensively reported. Globally, over 4 million deaths and 140 million disability-adjusted life years (DALYs) are attributable to EBW [17]. Cancer is among the leading causes of EBW-related deaths and DALYs [17]. Globally, approximately 481 thousand new cancer cases or 3.6% of all new cancer cases in adults are attributable to EBW [18]. Attributable cases were higher in countries with high and very high human development indices than low- and middle-income countries [18]. In Brazil, EBW was estimated to account for 15 thousand (4%) new cancer cases in 2012 and may cause over 29 thousand cancers (5%) in 2025 [5].

Several country-wide comparative risk assessment studies have quantified the potential impact of EBW on cancer outcomes [2, 5, 6, 18–20], including the economic burden of cancer [6, 21–27]. In the last decade, costs of cancer attributable to EBW were US$ 43 million in Thailand [25] and US$ 47 million in Ireland [24]. More recently, an Italian cost-of-illness study estimated that EBW was responsible for €13.3 billion in 2020, of which €0.33 were due to direct and indirect costs of cancer [21]. In Brazil, a few studies have quantified the costs of cancer attributable to EBW in Brazil [6, 22, 23]. Direct costs of cancer attributable to EBW in Brazil have ranged from US$ 30 million [6] to US$ 83 million [23]. Variability of results across studies reflects parameters used in the models, such as EBW prevalence, RR, time-lag, and cancer-related costs. Studies have used different methodological approaches to estimate PIF/PAF and included different types of cancers, currencies, and years of reference, which generate difficulties in comparing results.

Our study adds knowledge to the body of evidence by quantifying the future economic burden of cancer attributable to the increasing prevalence of EBW (US$ 62.8 million in 2030). We also projected potential savings in the federal direct healthcare costs of cancer in 2040 (US$ 10.3 to 26.6 million), considering counterfactual (alternative) scenarios of population-wide reductions in the BMI to be achieved in 2030. These results are relevant considering the increasing EBW epidemic worldwide and in Brazil.

Worldwide, the age-adjusted prevalence of obesity tripled between 1975 and 2016 [28]. In 2016, approximately 16% or 650 million adults had obesity [28]. In Brazil, the prevalence of obesity more than doubled in the last decade, increasing from 12.5% in 2008/09 to 25.9% in 2019 [1]. The projected prevalence of obesity in Brazil in 2030 suggest that three out of ten adults will be living with obesity. In addition, the prevalence of obesity classes II and III (≥35 kg/m^2^) should reach 10% in 2030 [Estivaleti JM, Guzman-Habinger J, Lobos J, Azeredo CM, Claro R, Ferrari G, Adami F, Rezende LFM, unpublished observations].

Hypotheses about the causes of the rising obesity epidemic globally are abundant. More than 30 years ago, Geoffrey Rose noted that etiology confronts two distinct issues: the determinants of individual cases and the determinants of incidence rate [29]. Notably, the determinants of incidence may not be the same as the determinants of individual cases [29]. Genetic predisposition, for instance, could explain where individuals are placed on the distribution of individual weights. However, they do not explain why the body mass has shifted towards heavier weights so rapidly in many populations [30]. The EBW determinants also incorporate several economic, social, and behavioral factors present in modern societies, including social and economic inequalities, an increased offer of high energy density foods, and sedentary behavior [31].

However, the rising share of ultra-processed foods in the food supply has been proposed as the most crucial cause of the obesity epidemic (determinant of incidence rate) [32, 33]. Changes in the food systems during the 70s have led to a rapid increase in food energy availability, particularly towards increased consumption of ultra-processed foods [30, 32, 33]. A recent systematic review of dietary surveys showed that the contribution of ultra-processed foods to total energy intake ranged from 16% in Colombia to 58% in the USA [34]. In Brazil, the contribution of ultra-processed foods to total energy intake increased from 14.3% in 2002 to 19.7% in 2017-18 [35]. Since these changes in the food system, the contribution of ultra-processed food in the population dietary patterns, as the whole population distribution of BMI, has shifted to the right, independent of age group and sex [30, 32]. These observations of period effect are strong signals that changes in the food systems are likely the primary determinant of the obesity epidemic [30]. Policies and public health interventions to reduce the consumption of ultra-processed foods are imperative to reverse the obesity epidemic. Examples include taxation, marketing restrictions, adequate labeling, environmental changes, and public health campaigns. These actions may have benefits for preventing diseases as well as economic benefits.

Our results showed that reductions in the prevalence of EBW through these actions might have economic benefits, mainly to cope with the increasing economic burden of cancer. For instance, we estimated that the highest potential impact on cancer costs was the reduction of BMI distribution to levels observed in 2008/09 (US$ 26.55 million), followed for the halting the rise in EBW prevalence, reaching the BMI distribution to levels observed in 2019 (US$ 24.24 million), eliminating obesity class II and III (US$ 12.71 million) and 1 kg/m^2^ reduction of BMI’ mean observed in 2019 among participants with BMI ≥ 25 kg/m^2^ (US$ 10.33 million). To achieve these (bold) scenarios, we need a comprehensive set of integrated actions, including health-promoting environments (e.g., healthy urban planning, community action, improving food and beverage supply, marketing restrictions, fiscal policies, creating and healthy and packaging and labeling improvements), communication for behavior change (e.g., health education actions, advice in health services, information campaigns) and change in the food system (e.g., intersectoral integrated actions).

To our knowledge, this is the first study aimed at quantifying the future economic burden of cancer attributable to EBW and the potential savings in federal direct healthcare costs of cancer by reducing population-wide BMI. We used an extensive, nationally representative survey of Brazilian adults, relative risks from meta-analysis, and nationwide registries of direct healthcare costs of cancers in the Brazilian Unified Health System. However, our study has some limitations. To estimate the PIF/PAF, we used information on self-reported weight and height, which may have led to misclassification bias of BMI categories. We assumed the pooled relative risks from meta-analysis, which did not include Brazilian cohort studies. Nevertheless, we performed the Monte Carlo simulation approach to incorporate the uncertainty of PIF/PAF estimates. We assumed at least a 10-year time lag between BMI and cancers and did not consider recurring events. Our counterfactual (alternative) scenarios considered the potential effect of the BMI reduction on increased cancer risk and its associated costs. We did not consider the adverse effects of the increase in the proportion of the adults in the BMI category < 25kg/m^2^, such as the increased risk of premenopausal breast cancer. On the other hand, in Brazil, only about 30% of new cases [4] and costs of breast cancer occur in premenopausal women, and this phenomenon (lower BMI increasing the cancer risk) has not been observed for other cancer types. Finally, our risk assessment model did not consider different lifetime BMI trajectories (e.g., cancer risk in people who lived overweight in childhood and young adulthood vs. those who gained weight in adulthood).Future studies may use other modeling approaches (e.g., multistate lifetable modeling of preventive interventions) to incorporate disease status and time dimension more appropriately [36].

## Conclusions

EBW may be responsible for 2.4% or US$ 62.8 million federal direct healthcare costs of cancer in 2030. We projected potential savings of approximately US$ 10.3 to 24.2 million in 2040 via population-wide reductions of BMI. Our findings may be helpful to support policies aimed at EBW and cancer prevention strategies in Brazil.

## Supplementary Information

**Additional file 1: Supplementary Material A.** World Cancer Research Fund/ American Institute for Cancer Research meta-analysis references. **Supplementary Material B.** Parameters considered in the macrosimulation model. **Supplementary Material C.** 10^th^ revision of the International Statistical Classification of Diseases and Related Health Problems codes. **Supplementary Material D.** Meta-analysis of prostate (advanced) cancer by incidence as outcome. **Supplementary Material E.** Meta-analysis of ovary cancer by incidence as outcome. **Supplementary Material F.** Relative risk of excess body weight-associated cancers per exposition category and sex. **Supplementary Material G.** Hyperlinks to publicly archived datasets.

## Data Availability

All data generated or analyzed during this study are included in this published article and its supplementary information files.

## Abbreviations

BMI: Body mass index
DALY: Disability-adjusted life years
EBW: Excess body weight
IBGE: Brazilian Institute of Geography and Statistics
INCA: Brazilian National Cancer Institute
ICD-10: 10^th^ Revision of the International Statistical Classification of Diseases and Related Health Problems
PAF: Population Attributable Fraction
PIF: Potential Impact Fraction
POF: Brazilian National Household Budget Survey
PNS: Brazilian National Health Survey
PPP: Purchasing power parity
RR: Relative risk
SIA-SUS: Ambulatory Information System of the Brazilian Unified Health System
SIH-SUS: Hospital Information System of the Brazilian Unified Health System
SUS: Brazilian Unified Health System
WCRF/AICR: World Cancer Research Fund/American Institute Cancer Research
