# Supplementary material for: The future costs of cancer attributable to excess body weight in Brazil, 2030-2040
Source: BMC Public Health. 2022 Jun 21;22:1236. doi: 10.1186/s12889-022-13645-4 (PMC9215059; doi:10.1186/s12889-022-13645-4)
Supplement: Supplementary file 1 — Additional file 1: Supplementary Material A. World Cancer Research Fund/ American Institute for Cancer Research meta-analysis references. Supplementary Material B. Parameters considered in the macrosimulation model. Supplementary Material C. 10th revision of the International Statistical Classification of Diseases and Related Health Problems codes. Supplementary Material D. Meta-analysis of prostate (advanced) cancer by incidence as outcome. Supplementary Material E. Meta-analysis of ovary cancer by incidence as outcome. Supplementary Material F. Relative risk of excess body weight-associated cancers per exposition category and sex. Supplementary Material G. Hyperlinks to publicly archived datasets. [file 12889_2022_13645_MOESM1_ESM.docx]

**Additional file 1 of the future costs of cancer attributable to excess body weight in Brazil, 2030-2040.**

**Supplementary Information**

**The future costs of cancer attributable to excess body weight in Brazil, 2030-2040**

L. F. M. Rezende et al.

| List of Supplementary Materials |
| --- |
| **Supplementary Material A:** World Cancer Research Fund/ American Institute for Cancer Research meta-analysis references |
| **Supplementary Material B:** Parameters considered in the macrosimulation model |
| **Supplementary Material C:** 10^th^ revision of the International Statistical Classification of Diseases and Related Health Problems codes |
| **Supplementary Material D:** Meta-analysis of prostate (advanced) cancer by incidence as outcome |
| **Supplementary Material E:** Meta-analysis of ovary cancer by incidence as outcome |
| **Supplementary Material F:** Relative risk of excess body weight-associated cancers per exposition category and sex |
| **Supplementary Material G:** Hyperlinks to publicly archived datasets |

**Supplementary Material A:** World Cancer Research Fund/ American Institute for Cancer Research meta-analysis references

| **Cancer type** | **Reference** |
| --- | --- |
| Breast | World Cancer Research Fund/ American Institute for Cancer Research/ Imperial College London. Continuous Update Project Team Members. World Cancer Research Fund International Systematic Literature Review. The Associations between Food, Nutrition and Physical Activity and the Risk of Breast Cancer, Jan. 2017. Available at <https://www.wcrf.org/dietandcancer> |
| Colorectal | World Cancer Research Fund/ American Institute for Cancer Research/ Imperial College London. Continuous Update Project Team Members. World Cancer Research Fund International Systematic Literature Review. The Associations between Food, Nutrition and Physical Activity and the Risk of Colorectal Cancer Sep. 2017. Available at <https://www.wcrf.org/dietandcancer> |
| Endometrium | World Cancer Research Fund/ American Institute for Cancer Research/ Imperial College London. Continuous Update Project Team Members. World Cancer Research Fund International Systematic Literature Review. The Associations between Food, Nutrition and Physical Activity and the Risk of Endometrial Cancer, Dec. 2012. Available at <https://www.wcrf.org/dietandcancer> |
| Esophagus | World Cancer Research Fund/ American Institute for Cancer Research/ Imperial College London. Continuous Update Project Team Members. World Cancer Research Fund International Systematic Literature Review. The Associations between Food, Nutrition and Physical Activity and the Risk of Oesophageal Cancer, Feb. 2015. Available at <https://www.wcrf.org/dietandcancer> |
| Gallbladder | World Cancer Research Fund/ American Institute for Cancer Research/ Imperial College London. Continuous Update Project Team Members. World Cancer Research Fund International Systematic Literature Review. The Associations between Food, Nutrition and Physical Activity and the Risk of Gallbladder Cancer, Dec. 2014. Available at <https://www.wcrf.org/dietandcancer> |
| Kidney | World Cancer Research Fund/ American Institute for Cancer Research/ Imperial College London. Continuous Update Project Team Members. World Cancer Research Fund International Systematic Literature Review. The Associations between Diet, Nutrition and Physical Activity and the Risk of Kidney Cancer Apr. 2015. Available at <https://www.wcrf.org/dietandcancer> |
| Liver | World Cancer Research Fund/ American Institute for Cancer Research/ Imperial College London. Continuous Update Project Team Members. World Cancer Research Fund International Systematic Literature Review. The Associations between Food, Nutrition and Physical Activity and the Risk of Liver Cancer, Jan. 2015. Available at <https://www.wcrf.org/dietandcancer> |
| Mouth, Pharynx and Larynx | World Cancer Research Fund/ American Institute for Cancer Research/ Imperial College London. Continuous Update Project Team Members. World Cancer Research Fund International Systematic Literature Review. The Associations between Food, Nutrition and Physical Activity and the Risk of Mouth, Pharynx and Larynx cancer, Apr. 2016. Available at https://www.wcrf.org/dietandcancer |
| Ovary | World Cancer Research Fund/ American Institute for Cancer Research/ Imperial College London. Continuous Update Project Team Members. World Cancer Research Fund International Systematic Literature Review. The Associations between Food, Nutrition and Physical Activity and the Risk of Ovarian Cancer, Dec. 2013. Available at <https://www.wcrf.org/dietandcancer> |
| Pancreas | World Cancer Research Fund/ American Institute for Cancer Research/ Imperial College London. Continuous Update Project Team Members. World Cancer Research Fund International Systematic Literature Review. The Associations between Food, Nutrition and Physical Activity and the Risk of Pancreatic Cancer, Oct. 2011. Available at <https://www.wcrf.org/dietandcancer> |
| Prostate | World Cancer Research Fund/ American Institute for Cancer Research/ Imperial College London. Continuous Update Project Team Members. World Cancer Research Fund International Systematic Literature Review. The Associations between Food, Nutrition and Physical Activity and the Risk of Prostate Cancer Sep.2014. Available at <https://www.wcrf.org/dietandcancer> |
| Stomach | World Cancer Research Fund/ American Institute for Cancer Research/ Imperial College London. Continuous Update Project Team Members. World Cancer Research Fund International Systematic Literature Review. The Associations between Food, Nutrition and Physical Activity and the Risk of Stomach Cancer, May 2015. Available at <https://www.wcrf.org/dietandcancer> |

**Supplementary Material B:** Parameters considered in the macrosimulation model

| **Parameter** | **Variable** | **Commentary** | **Source/ Year** |
| --- | --- | --- | --- |
| Nutritional status | Prevalence data and median body mass index (BMI) in adults ≥ 20 years who rely exclusively on the public health system. | Nutritional status evaluated considering the body mass index (BMI) obtained from measured weight and height.  We obtained the median BMI and prevalence rates for each excess body weight category and sex. Exposition categories: BMI < 25 kg/m² (Underweight and Normal); 25 ≤ BMI < 30 kg/m² (Overweight); 30 ≤ BMI < 35 kg/m² (Class I obesity); ≥ 35 kg/m² (Class II and III obesity). | National Household Budget Survey – POF 2008-2009 and National Survey of Health – PNS 2019 |
| Relative risk | Relative risk | We used the relative risks obtained from the linear dose-response meta-analysis. We converted these measures per increment of 1kg/m² and calculated the relative risks per exposition category considering the median BMI in each category. We stratified by sex, when available, cancer type, and exposition category.  Exposition categories: BMI < 25 kg/m² (Reference); 25 ≤ BMI < 30 kg/m²; 30 ≤ BMI < 35 kg/m²; ≥ 35 kg/m². | WCRF/AICR systematic review reports |
| Direct healthcare cost | Values of inpatient procedures related to cancers in adults ≥ 30 years paid by the federal government | Federal direct healthcare costs of inpatient procedures related to cancer approved for payment in the Brazilian Unified Health System. We stratified by sex and cancer type. | Hospital Information System of the Brazilian Unified Health System  (SIH-SUS)  2008-2019 |
| Direct healthcare cost | Values of outpatient procedures related to cancer in adults ≥ 30 years paid by the federal government | Federal direct healthcare costs of outpatient procedures related to cancer approved for payment in the Brazilian Unified Health System. We stratified by sex and cancer type. | Ambulatory Information System of the Brazilian Unified Health System  (SIA-SUS) 2008-2019 |

**Supplementary Material C:** 10^th^ revision of the International Statistical Classification of Diseases and Related Health Problems codes

| **Cancer type** | **ICD-10 code** |
| --- | --- |
| Breast | C50, C50.0, C50.1, C50.2, C50.3, C50.4, C50.5, C50.6, C50.8, C50.9 |
| Colorectal | C18, C18.0, C18.1, C18.2, C18.3, C18.4, C18.5, C18.6, C18.7, C18.8, C18.9, C19, C20 |
| Endometrium | C54, C54.0, C54.1, C54.2, C54.3, C54.8, C54.9 |
| Esophagus | C15, C15.0 C15.1, C15.2, C15.3, C15.4, C15.5, C15.8, C15.9 |
| Gallbladder | C23 |
| Kidney | C64, C65 |
| Liver | C22.0, C22.9 |
| Mouth, Pharynx and Larynx | C00.3, C00.4, C00.5, C00.8, C00.9, C01, C02, C02.0, C02.1, C02.2, C02.3, C02.4, C02.8, C02.9, C03, C03.0, C03.1, C03.9, C04, C04.0, C04.1, C04.8, C04.9, C05, C05.0, C05.1, C05.2, C05.8, C05.9, C06, C06.0, C06.1, C06.2, C06.8, C06.9, C09, C09.0, C09.1, C09.8, C09.9, C10, C10.0, C10.1, C10.2, C10.3, C10.4, C10.8, C10.9, C12, C13, C13.0, C13.1, C13.2, C13.8, C13.9, C14.0, C14.2, C32, C32.0, C32.1, C32.2, C32.3, C32.8, C32.9 |
| Ovary | C56 |
| Pancreas | C25, C25.0, C25.1, C25.2, C25.3, C25.4, C25.7, C25.8, C25.9 |
| Prostate | C61 |
| Stomach (cardia) | C16.0 |
| Stomach | C16, C16.0, C16.1, C16.2, C16.3, C16.4, C16.5, C16.6, C16.8, C16.9 |
| All invasive cancers | C00-C97 |

**Supplementary Material D:** Meta-analysis of prostate (advanced) cancer by incidence as outcome

We performed a new meta-analysis including only the studies with incidence outcome of the WCRF/AICR Systematic Literature Review (SLR) in the software STATA version 13 using *metan* command. We used random-effects model since it allows that the true effect size might differ from study to study.


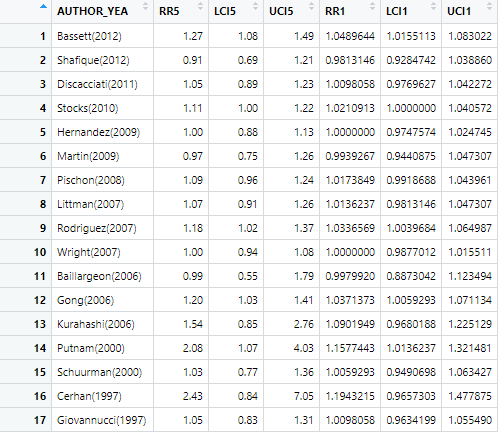


RR5: relative risk per increment of 5kg/m²

LCI5: lower 95% confidence interval of the relative risk per increment of 5kg/m²

UCI5: upper 95% confidence interval of the relative risk per increment of 5kg/m²

RR1: relative risk per increment of 1kg/m²

LCI1: lower 95% confidence interval of the relative risk per increment of 1kg/m²

UCI1: upper 95% confidence interval of the relative risk per increment of 1kg/m²


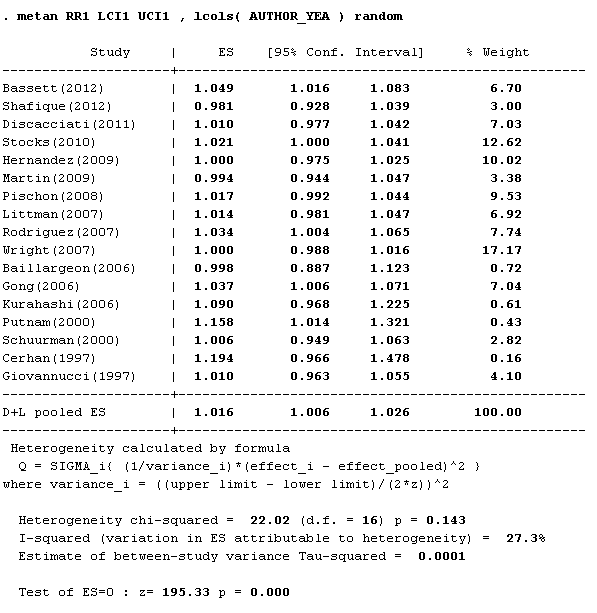


Abbreviations: ES, estimates (Relative Risk); CI


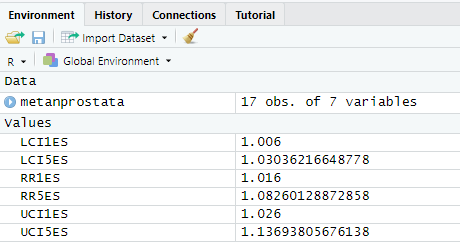


**Forest plot of the incidence studies of prostate (advanced) cancer.**

Abbreviations: ES, estimates (Relative Risk); CI, confidence interval

**Supplementary Material E:** Meta-analysis of ovary cancer by incidence as outcome

We performed a new meta-analysis including only the studies with incidence outcome of the WCRF/AICR Systematic Literature Review (SLR) in the software STATA version 13 using *metan* command. We used random-effects model since it allows that the true effect size might differ from study to study.


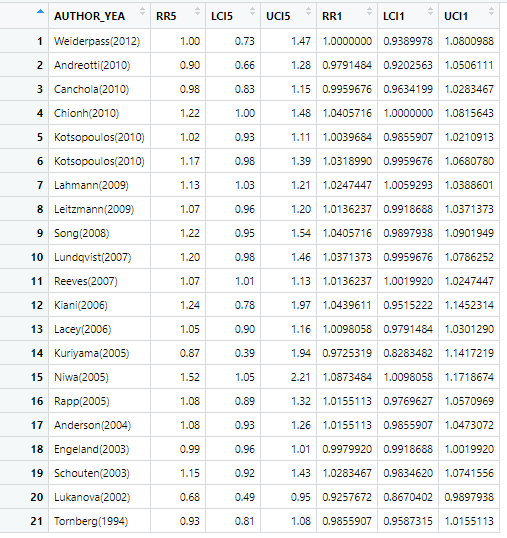


RR5: relative risk per increment of 5kg/m²

LCI5: lower 95% confidence interval of the relative risk per increment of 5kg/m²

UCI5: upper 95% confidence interval of the relative risk per increment of 5kg/m²

RR1: relative risk per increment of 1kg/m²

LCI1: lower 95% confidence interval of the relative risk per increment of 1kg/m²

UCI1: upper 95% confidence interval of the relative risk per increment of 1kg/m²


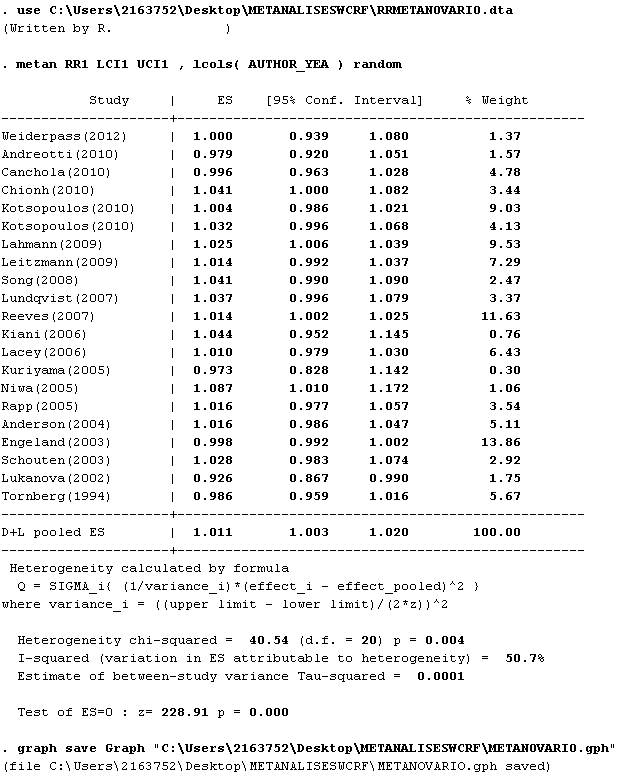


Abbreviations: ES, estimates (Relative Risk); Conf. Interval, confidence interval


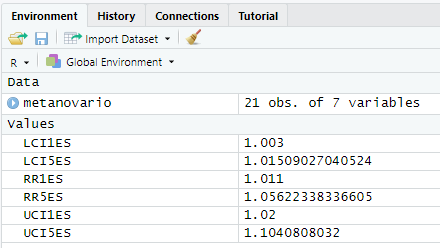


**Forest plot of the incidence studies of ovary cancer.**

Abbreviations: ES, estimates (Relative Risk); CI, confidence interval

**Supplementary Material F:** Relative risk of excess body weight-associated cancers per exposition category and sex.

| **Cancer type** | **Sex** | BMI < 25 kg/m² | 25 ≤ BMI < 30 kg/m² | 30 ≤ BMI < 35 kg/m² | BMI ≥ 35 kg/m² |
| --- | --- | --- | --- | --- | --- |
| Breast (post-menopausal) | F | 1.00 | 1.05 | 1.17 | 1.32 |
| Colorectal | F | 1.00 | 1.02 | 1.07 | 1.13 |
| Colorectal | M | 1.00 | 1.03 | 1.11 | 1.20 |
| Endometrium | F | 1.00 | 1.20 | 1.77 | 2.73 |
| Esophagus (adenocarcinoma) | F | 1.00 | 1.19 | 1.73 | 2.64 |
| Esophagus (adenocarcinoma) | M | 1.00 | 1.21 | 1.81 | 2.91 |
| Gallbladder | F | 1.00 | 1.10 | 1.34 | 1.67 |
| Gallbladder | M | 1.00 | 1.09 | 1.32 | 1.65 |
| Kidney | F | 1.00 | 1.13 | 1.44 | 1.92 |
| Kidney | M | 1.00 | 1.12 | 1.42 | 1.88 |
| Liver | F | 1.00 | 1.17 | 1.65 | 2.43 |
| Liver | M | 1.00 | 1.17 | 1.61 | 2.36 |
| Mouth, Pharynx and Larynx | F | 1.00 | 1.06 | 1.22 | 1.41 |
| Mouth, Pharynx and Larynx | M | 1.00 | 1.06 | 1.21 | 1.40 |
| Ovary | F | 1.00 | 1.02 | 1.08 | 1.15 |
| Pancreas | F | 1.00 | 1.04 | 1.14 | 1.27 |
| Pancreas | M | 1.00 | 1.05 | 1.18 | 1.34 |
| Prostate (advanced) | M | 1.00 | 1.03 | 1.11 | 1.21 |
| Stomach (cardia) | F | 1.00 | 1.10 | 1.34 | 1.67 |
| Stomach (cardia) | M | 1.00 | 1.09 | 1.32 | 1.65 |

Abbreviations: F, female; M, male; BMI, body mass index.

**Supplementary Material G:** Hyperlinks to publicly archived datasets.

| **Datasets** | **Hyperlink** |
| --- | --- |
| Brazilian National Household Budget Survey carried out in 2008/09 | [<https://www.ibge.gov.br/estatisticas/sociais/rendimento-despesa-e-consumo/9050-pesquisa-de-orcamentos-?=&t=microdados>familiares.html?=&t=microdados](https://www.ibge.gov.br/estatisticas/sociais/rendimento-despesa-e-consumo/9050-pesquisa-de-orcamentos-familiares.html?=&t=microdados) |
| Brazilian National Health Survey carried out in 2019 | <https://www.ibge.gov.br/estatisticas/sociais/saude/9160-pesquisa-nacional-de-saude.html?=&t=microdados> |
| Ambulatory Information System of the Brazilian Unified Health System | <ftp://ftp.datasus.gov.br/dissemin/publicos/siasus/200801_/dados> |
| Hospital Information System of the Brazilian Unified Health System | <ftp://ftp.datasus.gov.br/dissemin/publicos/sihsus/200801_/dados> |
